# Treatment of infective endocarditis caused by *Enterococcus faecalis* with a combination of penicillin G and ceftriaxone: a case report and literature review

**DOI:** 10.3389/fcvm.2025.1539372

**Published:** 2025-07-23

**Authors:** Li Jiang, Zhiqiang Lin, Shuifa Wu, Tingting Chen

**Affiliations:** ^1^ Department of Pharmacy, Quanzhou First Hospital Affiliated to Fujian Medical University, Quanzhou, Fujian, China; ^2^ College of Pharmacy, Fujian Medical University, Fuzhou, Fujian, China

**Keywords:** infective endocarditis, *Enterococcus faecalis*, ampicillin, penicillin, ceftriaxone

## Abstract

This report presents a case of Infective Endocarditis (IE) caused by *Enterococcus faecalis* (*E. faecalis*). The *E. faecalis* isolates were sensitive to ampicillin, penicillin G, and vancomycin. However, the outcome of anti-infection therapy was poor, and the patient was suspected to be allergic to vancomycin and ampicillin-sulbactam. This prompted various changes in antibiotic treatment regimens, with the patient eventually cured after administration of penicillin G combined with ceftriaxone (PC regimen). Literature was retrieved from the CNKI, Wanfang, Weipu, and PubMed databases to determine the efficacy of the PC regimen in the treatment of *E. faecalis*-induced IE. From the literature retrieved and our case study, there were only five reports of cases that had been treated with the PC regimen, with a mean age of (61.6 ± 17.2) years. The cases that had been previously reported in the literature involved patients of advanced age with complicated underlying diseases such as chronic obstructive pulmonary disease (COPD), atrial valve replacement, bladder carcinoma, and type 2 diabetes mellitus. The minimal inhibitory concentration (MIC) of ampicillin against the *E. faecalis* isolates from all five patients was <2 μg/ml, and all isolates showed susceptibility to penicillin G. All five patients were initially treated with other antimicrobial regimens but were eventually cured after switching to the PC regimen. In conclusion, ampicillin combined with ceftriaxone (AC regimen) can be substituted with the PC regimen for the treatment of IE caused by penicillin-susceptible *E.faecalis* when ampicillin is not available, when outpatient parenteral antimicrobial therapy (OPAT) with an AC regimen is not feasible, or when the patient is allergic to ampicillin.

## 1 Introduction

*Enterococci* are the third leading cause of Infective Endocarditis (IE), with 97% of enterococcal IE being caused by *E. faecalis* (1). The mortality rate of *enterococci*-induced IE is approximately 20%–40% (2). Penicillin, ampicillin, and vancomycin are antimicrobial agents that have bactericidal effects against streptococci but only bacteriostatic effects against *enterococci*. Therefore, a combined antibiotic regimen is recommended for treating enterococcal IE. For beta-lactam-susceptible *enterococci* infection, the American Heart Association (AHA) recommends a combination of ampicillin or penicillin G with gentamicin, or a combination of ampicillin with ceftriaxone (1). In contrast, the European Society of Cardiology (ESC) recommends a combination of ampicillin with gentamicin or ceftriaxone (3). In both cases, the AHA and ESC guidelines recommend dual-*β*-lactam therapy for the treatment of *E. faecalis*-induced IE. This study presents a clinical case of *E. faecalis*-induced IE that was cured after the treatment strategy switching to a PC regimen (Penicillin G combined with ceftriaxone). We also systemically reviewed the literature to evaluate the clinical efficacy and safety of the PC regimen, a novel therapeutic option for enterococcal IE. This study aimed to investigate the potential clinical application of the PC regimen in the treatment of *E. faecalis-*induced IE and to provide a reference for clinical treatment.

## 2 Case presentation

### 2.1 Patient admission

**Figure 1:**
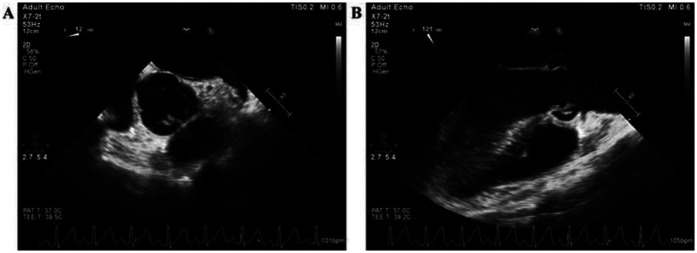
The image of transesophageal echocardiography before anti-infective therapy.

A 36-year-old male patient was admitted to the Department of Cardiovascular Surgery in our hospital on February 4, 2021, with a complaint of fever lasting for 4 days. The patient was diagnosed with rheumatic heart disease at another hospital more than 20 years prior to admission to our hospital. Color Doppler ultrasound showed emboli in the interphalangeal artery between the left index finger and middle finger, as well as in the dorsalis pedis artery. The results of the physical examination after admission were as follows: clear consciousness, T 39.4°C, P 115 beats/min, R 22 beats/min, and BP 115/84 mmHg. Breath sounds were clear on bilateral auscultation, and no dry or wet rales were heard. Heart rate was 115 beats per minute, paced heart rhythm and no murmur was heard in each valve auscultation area. The results of blood routine and PCT tests done on February 5 were: white blood cell 11.70 × 10^9^/L, neutrophil count 9.12 × 10^9^/L, lymphocyte count 1.61 × 10^9^/L, erythrocyte count 3.72 × 10^12^/L, hemoglobin 99 g/L, platelets 305 × 10^9^/L, and PCT 0.14 ng/ml. The results of coagulation screening were as follows: prothrombin time (PT) 17.0 s, international normalized ratio (INR) 1.54; Activated Partial Thromboplastin Time (APTT) 37.7 s, fibrinogen (FIB) 5.11 g/L, and D-dimer 0.63 mg/L. The results of complete set of biochemical testing done on February 5 were: total protein 62.3 g/L, albumin 28.9 g/L, total bilirubin 13.3 umol/L, alanine aminotransferase (ALT) 39 U/L, aspartate aminotransferase (AST) 28 U/L, glucose 4.96 mmol/L, blood urea nitrogen (BUN) 3.60 mmol/L, creatinine 72.0 umol/L, and plasma uric acid 154 umol/L. Autoantibodies for autoimmune hepatitis and TORCH screening were negative. Transesophageal echocardiography (TEE) showed that the right coronary valve had no coronary valve prolapse, multiple abnormal echogenicity (formation of vegetation), and severe aortic insufficiency (Figure 1).

After admission, the patient was diagnosed with a (1) infective endocarditis, and (2) moderate to severe aortic insufficiency; (3) aortic valve thickening without coronary valve prolapse, and (4) embolization of the dorsalis pedis artery, and (5) embolization of the interphalangeal artery between the left index finger and middle finger, and (6) fatty liver.

### 2.2 Duke-ISCVID criteria for infective endocarditis

On February 5, the TEE showed multiple abnormal echoes (redundancy formation) in the right coronary artery valve. blood cultures were performed on February 8, which showed six vials of aerobic and six vials of anaerobic cultures to be positive, and all of them cultured for *E. faecalis*. In addition, TEE showed that the patient had severe aortic regurgitation, a temperature of more than 38.0°C (39.4°C) on admission, and arterial embolization. Therefore, according to the Duke-International Society for Cardiovascular Infectious Diseases (Duke-ISCVID) Criteria for Infective Endocarditis, the patient met 2 major and at least 3 minor criteria for definite endocarditis (4).

### 2.3 Treatment process

**Figure 2:**
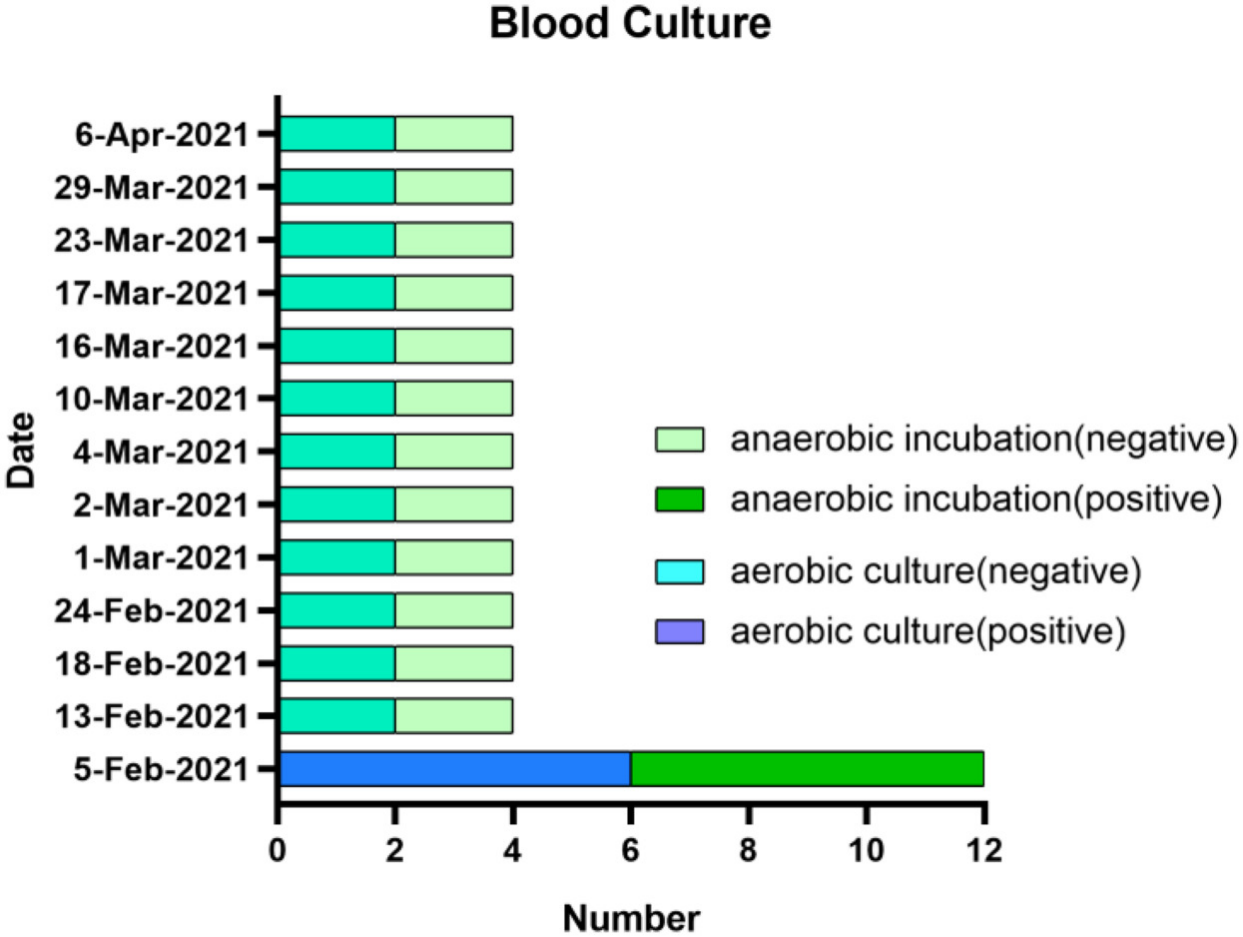
Blood culture status. *PC program, penicillin G (4.8 million units q6h iv) and ceftriaxone (2 g q12h iv).

Upon admission, cefotaxime (2 g q8h iv) was empirically given as the initial anti-infective regimen. On February 6, the patient was not febrile (37.2°C). On February 7, routine blood and CRP tests showed that white blood cell (WBC) count 10.40 × 10^9^/L, neutrophil count 8.39 × 10^9^/L, neutrophil ratio 80.70%, CRP 72.10 mg/L. On February 8, a blood culture revealed the growth of *E. faecalis* (6 bottles anaerobic culture, 6 bottles aerobic culture). In addition, automated susceptibility testing revealed that the *E. faecalis* isolates were resistant to amikacin and low-dose gentamicin but sensitive to high concentrations of ampicillin, penicillin G, and vancomycin (Table 1). On February 13, the treatment regimen was adjusted to intravenous administration of amoxicillin-clavulanate (Ratio 5:1, 1.2 g q8h). On February 13, blood cultures were negative (2 aerobic and 2 anaerobic), and 11 subsequent blood cultures were performed, all of which were negative (Figure 2). On February 19, the blood results showed that WBC count 12.85 × 10^9^/L, neutrophil count 9.96 × 10^9^/L and neutrophil ratio 77.6%. As the inflammatory markers were consistently higher than normal, treatment was switched to a combination of vancomycin (1 g q8h iv) and ceftriaxone (2 g qd iv). On February 23, the patient's temperature was normalized (36.8°C), inflammatory indicators improved (WBC count 8.51 × 10^9^/L, neutrophil count 6.07 × 10^9^/L and neutrophil ratio 71.3%), and he was changed to vancomycin monotherapy to continue treatment. On February 28, the patient was again febrile (37.9°C) with normal inflammatory markers (WBC count 6.11 × 10^9^/L, neutrophil count 3.98 × 10^9^/L and neutrophil ratio 65.2%). In addition, craniocerebral magnetic resonance imaging (MRI) performed on February 27 and March 2 revealed multiple hemorrhagic infarctions in the left parietal and right frontal lobes. Evacuation of the aortic valve vegetation and replacement with a mechanical valve were performed on March 4. After the operation, the patient continued to receive vancomycin as anti-infection therapy, enoxaparin sodium as anticoagulation therapy, and symptomatic and supportive treatment, including cardiotonic, diuretic, and postoperative nutritional support.

**Table 1:** The automated susceptibility testing (*Enterococcus faecalis*).

Antimicrobial	Minimum inhibitory concentration	Sensitivity	Reporting of results
Gentamicin Screening Test	≤500 μg/ml	Susceptible	S
Amikacin	16 μg/ml	Resistance	R
Gentamicin	≤1 μg/ml	Resistance	R
Tobramycin	4 μg/ml	Resistance	R
Ampicillin	≤2 μg/ml	Susceptible	S
Penicillin	26 mm (Kirby-Bauer test)	Susceptible	S
Amoxicillin-Clavulanic acid	≤1/0.5 μg/ml	Susceptible	S
Teicoplanin	≤1 μg/ml	Susceptible	S
Vancomycin	≤1 μg/ml	Susceptible	S
Clindamycin	>2 μg/ml	Resistance	R
Erythromycin	>4 μg/ml	Resistance	R
Fusidic acid	>8 μg/ml	Resistance	R
Linezolid	25 mm (Kirby-Bauer test）	Susceptible	S
Ciprofloxacin	1 μg/ml	Susceptible	S
Rifampicin	≤0.5 μg/ml	Susceptible	S
Tetracycline	>8 μg/ml	Resistance	R

**Figure 3:**
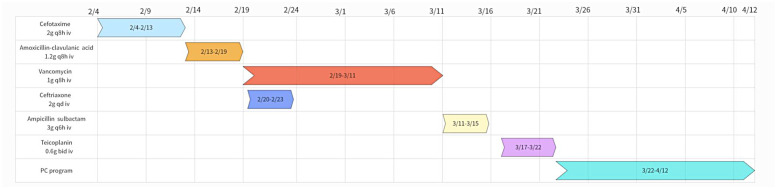
Anti-infection program.

**Figure 4:**
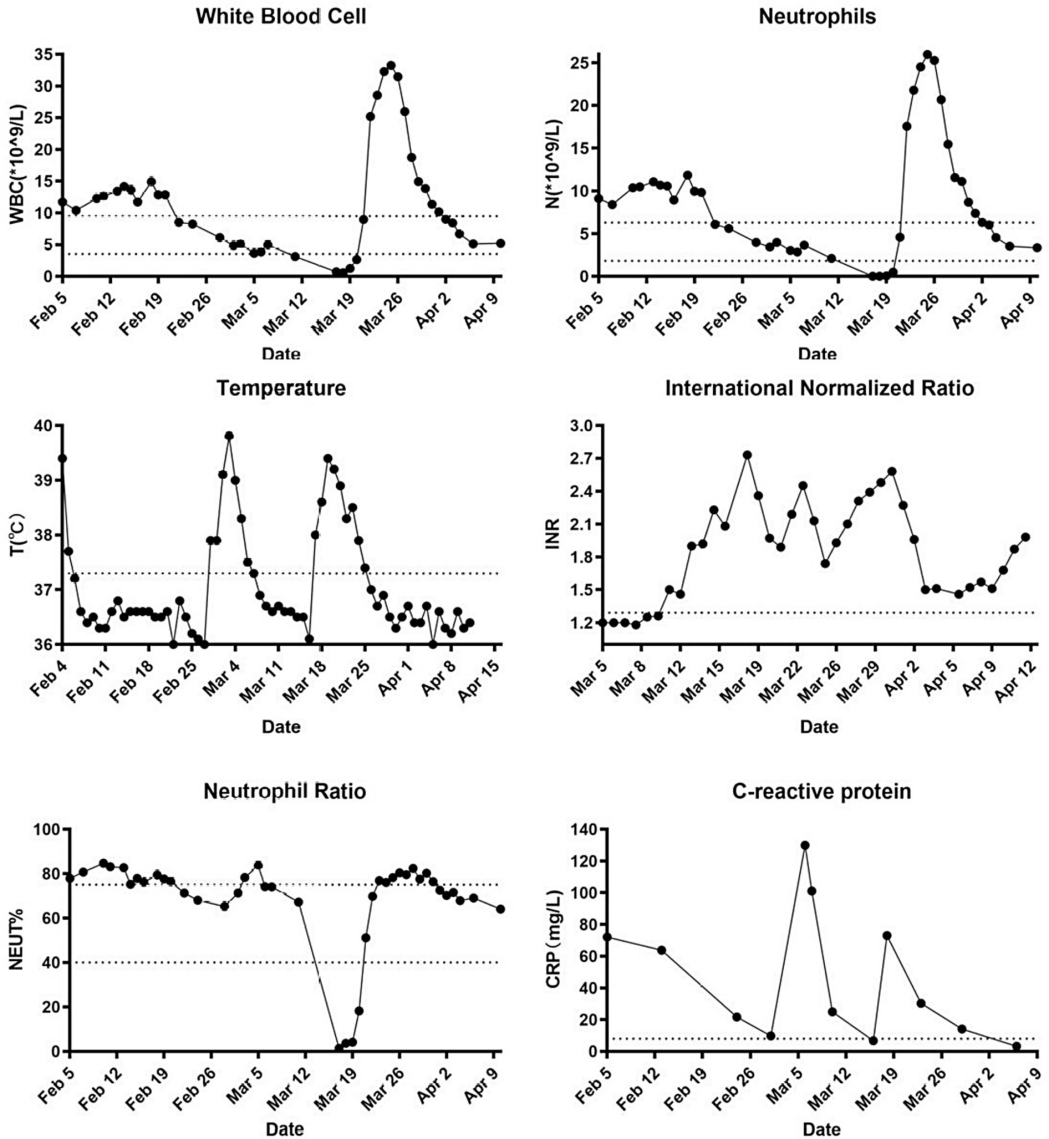
Laboratory indicators and temperature.

On March 7, the patient's temperature returned to normal (37.3°C), and the inflammatory indicators were not elevated (WBC count 4.93 × 10^9^/L, neutrophil count 3.65 × 10^9^/L, neutrophil ratio 74.0% and CRP 101.11 mg/L). On March 11, the patient developed a red, diffuse, and pruritic rash on the upper extremities and lower back. On March 12, the patient was considered allergic to vancomycin and discontinued. The patient was subsequently switched to intravenous administration of ampicillin-sulbactam (ratio 2:1, 3 g, q6h), as well as dexamethasone and loratadine, for anti-allergic treatment. On March 15, the patient developed a severe rash that extended to the thoracolumbar region as well as the upper and lower extremities. Ampicillin sulbactam was discontinued because it could not be ruled out that ampicillin sulbactam may have caused or exacerbated the rash. On March 17, the anti-infective regimen was changed to intravenous ticlopidine (0.6 g bid). Meanwhile, routine blood results showed that the patient had a WBC count 0.71 × 10^9^/L, neutrophil count 0.01 × 10^9^/L and neutrophil ratio 1.4%, which were significantly lower than normal values. Granulocyte-macrophage colony-stimulating factor (GM-CSF) was clinically administered to stimulate WBC production (150 μg, subcutaneous injection). After 2 days, the rash improved but remained febrile (39.4°C), and laboratory markers remained below normal. Accordingly, anti-infection, leukocyte stimulation, cardiotonic, diuretic, anticoagulant, and symptomatic treatments were continued. On March 22, the patient remained febrile (38.3°C) with elevated inflammatory markers (WBC count 25.18 × 10^9^/L, neutrophil count 17.57 × 10^9^/L and neutrophil ratio 69.8%). After a multidisciplinary consultation involving cardiovascular surgery, infectious diseases, neurology, intensive care medicine, and pharmacy, ticlopidine and GM-CSF were discontinued, and the anti-infective regimen was changed to a combination of penicillin G (4.8 million units q6h iv) and ceftriaxone (2 g q12h iv). On March 26, the patient's temperature returned to normal (37.0°C). On April 3, the patient's inflammatory indexes were normalized (WBC count 8.4 × 10^9^/L, neutrophil count 6.01 × 10^9^/L and neutrophil ratio 71.5%). On April 12, the patient was discharged from the hospital because his vital signs were stable and the infection had been brought under control. The course of oral amoxicillin was continued for 7 days after discharge; the full course of treatment lasted approximately 65 days. After outpatient follow-up, the patient was cured without recurrence. The patient's medications are shown in Figure 3, and specific laboratory parameters and temperature changes over time are shown in Figure 4.

## 3 Literature review

To investigate the reliability of the PC regimen for the treatment of *E.faecalis-*induced IE, the relevant literature was retrieved from the CNKI, Wanfang, Weipu, and PubMed databases. The search terms used were “infective endocarditis”, “Enterococcus faecalis”, “penicillin” and “ceftriaxone”. The search time limit was set from the establishment of each database until March 2025. Two researchers conducted the independent searches. Once duplicates were removed, the researchers identified studies that were eligible for analysis by examining the titles and abstracts of every record, followed by full-text reviews. Any disagreement between the reviewers was resolved by discussion with arbitration by a third reviewer when required. Literature involving cases with complete clinical data and those who had received the PC regimen for the treatment of *E. faecalis-*induced IE were included in the present study. The exclusion criteria were as follows: literature review, repeated published literature, and literature with incomplete clinical data [including demographic data, drug dosage, duration of therapy, and minimum inhibitory concentrations (MIC) of ampicillin and penicillin G].

A total of five patients were included in the study and their mean age was 61.6 ± 17.2 years. Unlike the case reported in our hospital, the four patients reported in the literature were of advanced age with complicated underlying diseases, namely chronic obstructive pulmonary disease (COPD), atrial valve replacement, bladder carcinoma, and type 2 diabetes mellitus. The MIC value of ampicillin against *E. faecalis* isolated from all five patients was <2 μg/ml. In addition, the *E. faecalis* isolates from the five patients showed sensitivity to penicillin G, of which two isolates had an MIC of 4 μg/ml and one isolate had an MIC of 2 μg/ml, whereas data for the remaining two isolates were not available. Two patients switched to the PC regimen because of the inconvenience of continuing treatment with the Ampicillin and ceftriaxone (AC regimen) in the outpatient clinic after discharge. Persistent bacteremia forced the AC regimen of another patient to be replaced by the PC regimen. The PC regimen was ultimately adopted in the fourth patient because of ototoxicity induced by the prolonged use of penicillin G combined with gentamicin (penicillin plus gentamicin, referred to as the PG regimen). Treatment for the patient admitted to our hospital was switched to the PC regimen because of suspected vancomycin- and ampicillin-sulbactam-induced allergic reactions. All five patients were eventually cured after switching to the PC regimen. For further details, see Table 2.

**Table 2:** Summary of five patients with Enterococcus faecalis infective endocarditis treated with combination of penicillin G and ceftriaxon.

Prior therapy (days)	Amp MIC (mg/L)	PCG MIC (mg/L)	Dose of PCG	Treatment duration	Oral transition	Outcome
10 (TZP then AC) (24)	<2	2	24 million units/day	6 weeks	None	Cured
3 (AC) (24)	<2	4	24 million units/day	7 weeks	Amoxicillin	Cured
7 (AC) (24)	<2	NA	24 million units/day	6 weeks	None	Cured
32 (PG) (24)	<2	4	24 million units/day	8 weeks	Amoxicillin	Cured
46 (CTX, AMC, VAN, CRO, SAM)^a^	<2	NA	19.2 million units/day	4 weeks	Amoxicillin	Cured

AC, Ampicillin + Ceftriaxone; PG, Penicillin + Gentamicin; Amp, ampicillin; PCG, penicillin; TZP, piperacillin/tazobactam; CTX, cefotaxime; AMC, amoxicillin and clavulanate potassium; VAN, vancomycin; CRO, cefatriaxone; SAM, ampicillin sulbactam; COPD, chronic obstructive lung disease; AVR, atrial valve replacement; DM, diabetes mellitus; MIC, minimal inhibitory concentration.

^a^ Cases in this study.

## 4 Discussion

### 4.1 Risk factors of enterococcal IE

Patients with enterococcal IE tend to be elderly individuals (5). In addition, patients with cancer, aortic valve vegetation, and primary abdominal or urogenital infections have a higher risk of developing enterococcal IE than those with other types of IE (6). Moreover, the increase in the number of abdominal and urogenital surgeries as well as the incidence of enterococcal catheter-associated bacteremia has led to an increase in the incidence of hospital-acquired enterococcal IE (5). As shown in Table 2, the four patients reported in the retrieved literature were all older than 50 years and had underlying diseases. However, it is worth noting that the patient in our case report was a young man without any underlying disease, which distinguishes this case from the others.

### 4.2 Mechanisms of action and clinical application of dual β-lactam therapy

To the best of our knowledge, β-lactam antibiotics are not bactericidal against *enterococci*. However, antibiotics that disrupt the synthesis of bacterial cell walls facilitate the intracellular uptake of aminoglycosides, which exert bactericidal activity by inhibiting protein synthesis (1). Thus, ampicillin, penicillin G, and vancomycin exhibit synergistic effects when used in combination with gentamicin. Although aminoglycoside-containing regimens have traditionally been considered the standard treatment regimen for enterococcal IE, the availability of fewer nephrotoxic agents and the rising incidence of high-level aminoglycoside resistance have led to the need for novel therapeutic strategies. Dual-β-lactam regimens have emerged as attractive candidates for severe *E. faecalis* infections because they are well tolerated during long-term use and have favorable side-effect profiles. The *in vitro* synergistic efficacy of a dual-β-lactam combination against *E. faecalis* was first demonstrated in 1995 (7). The MIC50 of amoxicillin against *E. faecalis* decreased from 0.5 mg/ml to 0.06 mg/ml when combined with cefotaxime at a concentration of 4 μg/ml (7). The MIC50 of cefotaxime against *E. faecalis* decreased from 256 μg/ml to 1 μg/ml when combined with amoxicillin at a concentration of 0.06 μg/ml (7). The observed synergistic efficacy between the two compounds was attributed to the complete saturation of penicillin-binding proteins (PBPs) 2 and 3 by cefotaxime and the partial saturation of PBPs 4 and 5 by amoxicillin, which theoretically disrupts further synthesis of the bacterial cell wall and induces bacterial autolysis (7). However, it should be noted that this combination regimen may only be useful in cases of *E. faecalis-*induced IE because the major PBPs of *enterococcus faecium* (*E. faecium*) are types 1 and 5. Several animal experiments and case reports have further verified the efficacy of combining ampicillin and cefotaxime, specifically for treating enterococcal IE (8). There is evidence supporting the clinical application of the AC regimen, since the AC regimen is the only dual beta-lactam regimen recommended by major guidelines (1, 3). Four observational clinical studies verified the safety and effectiveness of the AC regimen for the treatment of enterococcal IE (8). One study examined the therapeutic efficacy of ampicillin and ceftriaxone alone, whereas the other three studies compared the AC regimen to the current standard of ampicillin plus gentamicin. The clinical cure rate of 2 g ampicillin every 4 h in combination with 2 g ceftriaxone every 12 h is similar to that of ampicillin in combination with gentamicin (8). Besides, the AC regimen is well tolerated, with minimal risk of renal failure (0%–33% vs. 20%–65%) (8). The antimicrobial activity of ampicillin-sulbactam against *E. faecalis* is not stronger than that against ampicillin because changes in bacterial PBPs are thought to be due to penicillin resistance in *enterococci* and not due to penicillinases (9). Our patient was treated with amoxicillin-clavulanate and ampicillin-sulbactam because amoxicillin and ampicillin were unavailable.

The patient in this case was initially empirically treated with cefotaxime monotherapy, which was later changed to amoxicillin clavulanic acid monotherapy. Neither regimen was consistent with the combination regimen recommended by major guidelines. Also, laboratory tests showed elevated inflammatory markers, which implied poor infection control. The drug sensitivity report came back with results showing Enterococcus faecalis and resistance to low doses of gentamicin. Guidelines recommend that patients should be tested for resistance to high doses of gentamicin rather than low doses, but gentamicin was not chosen for treatment due to limited hospital conditions and the unavailability of gentamicin in the hospital. The clinical regimen was changed to vancomycin in combination with ceftriaxone and the infection was controlled. Subsequently, the patient underwent surgery for evacuation of the aortic valve vegetation and replacement of the aortic valve with a mechanical valve. The therapeutic course of enterococcal IE is six consecutive weeks, yet patients are prone to vancomycin-induced ototoxicity or nephrotoxicity during the prolonged course of vancomycin-containing regimens. Therefore, vancomycin should be avoided in the treatment of infections caused by penicillin-susceptible E. faecalis. This is one of the reasons why vancomycin was discontinued in this case after the infection was controlled. The most important reason is that the patient had a serious adverse reaction. The patient in this case applied vancomycin and ampicillin sulbactam prior to the development of neutropenia, which is thought to be the cause of neutropenia. However, after the application of vancomycin and before the administration of ampicillin sulbactam, the patient's neutrophils had already begun to decrease, whereas amoxicillin clavulanic acid, which had been applied much earlier, did not lead to neutropenia, and it was therefore considered that the neutropenia was most likely due to vancomycin. A Naranjo score of 4 was performed, indicating that the adverse drug reaction was most likely due to vancomycin. In conclusion, it is considered more likely that the patient's rash was caused by vancomycin rather than ampicillin sulbactam, but the possibility of an exacerbation of the adverse reaction by ampicillin sulbactam cannot be excluded. In addition, because the blood concentrations of vancomycin were not measured during treatment, it cannot be excluded that the occurrence of adverse reactions was due to vancomycin concentrations outside the therapeutic concentration range.

### 4.3 The applicable populations for PC regimen

In addition to the cases shown in Table 1, Tritle et al. (10) reported three cases that were cured using the PC regimen. However, the authors did not provide detailed information about each case, leading to the exclusion of three cases from the present study. Clinical *E. faecalis* isolates often show sensitivity to ampicillin *in vitro*. The MIC values for ampicillin and penicillin exhibited good concordance *in vitro*, and the majority of *E. faecalis* isolates showed cross-susceptibility to imipenem and penicillin (11). Ampicillin-susceptible, but penicillin-resistant (ASPR) *E. faecalis* strains have been isolated worldwide. A study also conducted by Tritle BJ et al. (10) showed that ASPR *E. faecalis* accounted for only 2.06% of *E. faecalis* isolates (*n* = 669). Additionally, a study conducted by Kim et al. (12) revealed that the MIC values of ampicillin against ASPR *E. faecalis* were 1-to 3-fold-lower than those of penicillin against ASPR *E. faecalis*, consistent with previous findings (11, 13). The ASPR *E. faecalis* isolates accounted for 22.7% (67 of 295 strains) in another observational study (12). The 30-day mortality rate of bloodstream infections（BSIs）caused by ASPR *E. faecalis* was 26.9%, which was >2-fold-higher than BSIs that of penicillin-susceptible *E. faecalis* (12.3%). For patients with ASPR, *E. faecalis*-induced BSIs and ampicillin- or piperacillin-containing regimens could result in anti-infective therapeutic failure, even though the *E. faecalis* strains exhibited susceptibility to ampicillin *in vitro* (12). There is no evidence to support the use of ampicillin over penicillin despite its stronger antibacterial activity *in vitro* (6). Therefore, the susceptibility of *E. faecalis* isolates to penicillin G and ampicillin should be determined prior to clinical application. The PC regimen was appropriate for patients who were sensitive to penicillin G.

IE often requires a prolonged treatment course and outpatient parenteral antimicrobial therapy (OPAT) appears to be an attractive option for patients with IE (14–17). β-lactam antimicrobials are known to display time-dependent antimicrobial activity, with only a brief postantibiotic effect because of their short half-lives. For some refractory infections, β-lactam antimicrobials are best administered via continuous infusions. Ampicillin is a β-lactam antimicrobial agent with a short half-life that is unstable at room temperature, making it unsuitable for continuous infusion. Ampicillin and penicillin G solutions remained stable for 8 h and 2 days, respectively, at 25°C (18). Ampicillin is administered up to six times per day in patients with normal renal function (19). Moreover, ampicillin cannot be mixed with ceftriaxone, necessitating two additional doses of intravenous ceftriaxone per day (20). Thus, the need for eight doses per day limits the use of ampicillin-containing regimens for *E. faecalis-*induced IE during OPAT (21). The recommended use of penicillin G by the AHA and ESC guidelines is fractionated dosing or continuous intravenous infusion, whereas only fractionated dosing is recommended for ampicillin (1, 3). The drawback of frequent intermittent infusions is the possibility of dose omission due to errors by nurses, doctors or pharmacists (22). The absence of patients and difficulty in securing vascular access may result in delayed drug administration. Moreover, frequent intermittent infusion may disrupt sleep and nighttime rest, resulting in a major negative impact on the patient's quality of life (23). Therefore, penicillin G is more suitable for continuous intravenous infusion. Although the MIC of ampicillin against *E. faecalis* is often one-to three-fold lower than that of penicillin G (12), substituting ampicillin with penicillin G is an attractive novel treatment option. From this perspective, the administration of ceftriaxone twice per day and penicillin G continuously per day reduces the number of injections from eight to three, making the PC regimen more feasible for OPAT. Two patients in the literature were switched from ampicillin to penicillin G at the time of OPAT initiation (24). It is noteworthy that cases of continuous intravenous infusion of ampicillin have also been recently reported (25, 26), but further clinical validation is needed.

In our case, although vancomycin was the most likely cause of the rash, we cannot exclude the possibility of ampicillin-sulbactam-induced allergy. After a negative penicillin G skin test, no allergic reaction was observed when the patient was switched to penicillin G. The beta-lactam ring structure is common to all beta-lactam antibiotics, but the most significant allergenic determinant of aminopenicillin is the R1 side chain (27). Among individuals with IgE-mediated aminopenicillin allergies, some are selectively allergic to aminopenicillins and show good tolerance to benzylpenicillin, whereas others respond to the allergenic determinants of benzylpenicillin. In both the American practice parameters (28) and the European Network for Drug Allergy (ENDA) guidelines (29–31), skin testing with penicilloyl-polylysine (PPL) and the minor determinant mixture (MDM) represents the first-line method for evaluating hypersensitivity reactions to β-lactams. Selective reactions to aminopenicillin were observed in 42.1% of 920 immediate allergies patients (26). in contrast, non-selective reactions accounted for 57.9% (32). Only 14% of patients (7/51) who developed immediate allergic reactions to aminopenicillins responded to allergenic determinants of benzylpenicillin (33). Research concerning allergy skin testing was performed on 157 subjects who displayed a delayed-type allergy to penicillin reagents, mostly aminopenicillins (34). The results of the study showed that pure side-chain sensitization occurred in 60% of the study participants. More importantly, these patients were negative for benzylpenicillin, benzylpenicilloyl-poly-L-Lysin, and a minor determinant mixture (34). In brief, the penicillin G skin-prick test can be performed in patients allergic to ampicillin, and penicillin G can be used to treat *E. faecalis-*induced IE in patients with negative skin prick test results.

## 5 Conclusion

In summary, the PC regimen can be used for the treatment of IE caused by penicillin-susceptible *E. faecalis* despite the lack of guideline endorsement. Consequently, the AC regimen can be substituted with the PC regimen when ampicillin is not available, OPAT therapy with AC is not feasible, or the patient is allergic to ampicillin. Furthermore, future larger studies with more cases are essential to validate the present findings and shed more light on the therapeutic potential of the PC regimen. In this sense, we hope that our results will encourage reassessment of the reliability of the PC regimen for *E. faecalis*-induced IE.

## Data Availability

The original contributions presented in the study are included in the article/Supplementary Material, further inquiries can be directed to the corresponding authors.
